# Photothermal Off-Resonance Tapping for Rapid and Gentle Atomic Force Imaging of Live Cells

**DOI:** 10.3390/ijms19102984

**Published:** 2018-09-30

**Authors:** Adrian P. Nievergelt, Charlène Brillard, Haig A. Eskandarian, John D. McKinney, Georg E. Fantner

**Affiliations:** 1 Laboratory for Bio- and Nano-Instrumentation, Swiss Federal Institute of Technology Lausanne (EPFL), 1015 Lausanne, Switzerland; adrian.nievergelt@epfl.ch (A.P.N.); charlene.brillard@epfl.ch (C.B.); alex.eskandarian@epfl.ch (H.A.E.); 2 UPKIN, Swiss Federal Institute of Technology Lausanne (EPFL), 1015 Lausanne, Switzerland; john.mckinney@epfl.ch

**Keywords:** high-speed atomic force microscopy, photothermal off-resonance tapping, live cell imaging, antimicrobial peptide, thrombocytes, bacterial imaging, cell lysis

## Abstract

Imaging living cells by atomic force microscopy (AFM) promises not only high-resolution topographical data, but additionally, mechanical contrast, both of which are not obtainable with other microscopy techniques. Such imaging is however challenging, as cells need to be measured with low interaction forces to prevent either deformation or detachment from the surface. Off-resonance modes which periodically probe the surface have been shown to be advantageous, as they provide excellent force control combined with large amplitudes, which help reduce lateral force interactions. However, the low actuation frequency in traditional off-resonance techniques limits the imaging speed significantly. Using photothermal actuation, we probe the surface by directly actuating the cantilever. Due to the much smaller mass that needs to be actuated, the achievable measurement frequency is increased by two orders of magnitude. Additionally, photothermal off-resonance tapping (PORT) retains the precise force control of conventional off-resonance modes and is therefore well suited to gentle imaging. Here, we show how photothermal off-resonance tapping can be used to study live cells by AFM. As an example of imaging mammalian cells, the initial attachment, as well as long-term detachment, of human thrombocytes is presented. The membrane disrupting effect of the antimicrobial peptide CM-15 is shown on the cell wall of *Escherichia coli*. Finally, the dissolution of the cell wall of *Bacillus subtilis* by lysozyme is shown. Taken together, these evolutionarily disparate forms of life exemplify the usefulness of PORT for live cell imaging in a multitude of biological disciplines.

## 1. Introduction

The mechanical nature of atomic force microscopy (AFM) makes it a powerful complementary technique to optical imaging for live-cell experiments, because it can offer nanometer resolution [1,2] as well as mechanical information of the sample [3,4,5]. While there are a wide range of imaging modalities available for AFM, only a subset is usable for live cell imaging. The main difficulties in observing living cells is that they are either very soft or not well attached to the underlying substrate [6,7]. Classical AFM modes like amplitude modulation or contact mode tend to require the sample to be well attached when working in liquid due to large interaction forces, both normal [8,9] as well as lateral [10]. Additionally, neither of these modes on their own can provide reliable mechanical information on the sample [11]. Often, force-distance-based modes provide both better force control as well as less lateral interaction during scanning, although usually at the cost of imaging speed when compared to resonant modes [12].

Force volume, where individual force ramps are done in multiple points of the sample [13,14], provides the best force control, as well as reliable mechanical property measurements. However, the imaging rate, in the order of tens of minutes to hours per image, is by far too slow to observe dynamic effects on cells. Off-resonant modes (pulsed force mode, PeakForce, jumping mode, hybrid, QI mode), on the other hand, modulate the tip–sample distance periodically at a frequency much lower than the resonance frequency of the cantilever to obtain force interactions at the modulation rate [15,16]. Previously, off-resonant modes have been applied with great success to a wide range of problems in molecular and cell biology, such as molecular recognition [17,18,19], cell mechanics [20,21], and host–pathogen interaction [22]. The maximum force during such an interaction is then used as the main feedback variable during scanning. In addition to increasing the image acquisition speed, these modes are well suited to obtain mechanical contrast, since the resulting periodic force interactions can be analyzed in real time. Furthermore, off-resonance modes also tend to be easier to use, especially in liquid, since they can be made immune to set-point drift. In most resonant modes, the free amplitude becomes unobservable once in feedback. Since the amplitude is being actively controlled for, the observed amplitude will always correspond to the set-point, as long as the set-point amplitude is smaller than the free amplitude and the piezo is in range. However, the free amplitude can change almost arbitrarily. The free amplitude can therefore only be checked by periodically withdrawing from the surface. In contrast, the interaction in off-resonant modes can be fully observed in every cycle, since the cantilever is disengaged from contact for most of the measurement cycle, allowing for the establishment of a per-cycle tip–sample force-free baseline value. These properties make off-resonance modes an excellent choice to study live cells [2,23].

However, even at the improved imaging speeds, it often takes on the order of several minutes to acquire an image, especially on difficult samples like live cells [24].

**Figure 1:**
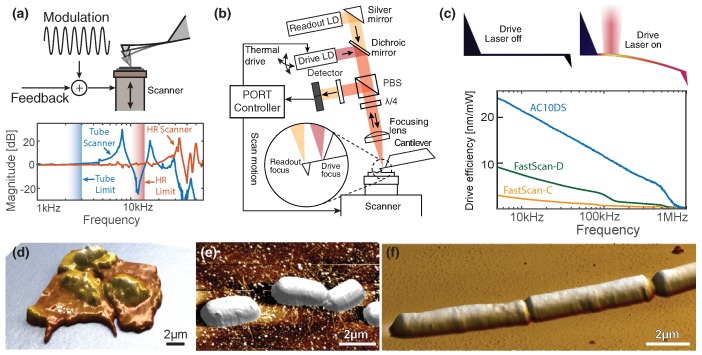
Working principle and implementation of photothermal off-resonance tapping (PORT). (**a**) Conventionally, off-resonance modes use the axial motion of the scanner to periodically probe the surface in a controlled fashion [31]. The resonances of those scanners limit the probing frequency to about 2 kHz for tube scanners and to about 12 kHz for a high-rate (HR) scanner. (**b**) Schematic of PORT setup and operation: the cantilever is driven with a secondary driving laser (685 nm) while a dedicated PORT controller is used to extract the tip–sample interaction from the deflection of the readout laser (635 nm) off the backside of the cantilever. (**c**) The heating induced by the drive laser (thermal distribution along the cantilever, indicated top right) causes a differential expansion in the cantilever, which leads to controlled bending. Smaller cantilevers are more suited to PORT, due both to increased driving efficiency as well as higher resonance frequency. Examples of live cells scanned in PORT are (**d**) human thrombocytes, (**e**) Gram-negative bacteria (*Escherichia coli*), as well as (**f**) Gram-positive bacteria (*Bacillus subtilis*). The *z*:*x*-*y* ratio in (**d**–**f**) is 1:1.

The speed limit in classical off-resonance modes stems from the fact that the tip–sample modulation frequency has to be kept significantly below the first *z*-resonance of the scanner in order to produce a controlled motion (see Figure 1a). To bypass the inertial effect which causes the scanner resonances, we actuate the cantilever directly using a laser beam (Figure 1b). By this method, generally known as photothermal excitation [25,26,27,28,29], the mass which needs to be actuated is reduced to only the cantilever and the tip (Figure 1c). It is therefore possible to increase the rate at which the surface is interrogated by over 2 orders of magnitude, while maintaining the ability to use large amplitudes and image with a controlled force. Previously, we have shown how this technique, called photothermal off-resonance tapping (PORT), can be used to measure the self-assembly of proteins in real time [30]. Here we show how the same technique enables robust imaging of living cells at substantially higher speeds than traditionally possible (Figure 1d–f). One of the key strengths of PORT is the ability to use large oscillation amplitudes of easily more than 50 nm, even when operating in liquid, while at the same time maintaining a small force interaction. Such large amplitudes reduce the probability that a large change in sample height will quench the whole oscillation. In that occurrence, very large lateral force interactions are expected while the tip stays in contact with the sample for a prolonged amount of time. Such large changes in height are common with big samples, such as living cells. As a consequence, both bacteria and eukaryotes alike can be maintained in physiological conditions and rapid events measured. Due to the slow time scales involved, traditional time-lapse AFM techniques used to study cells [24] often do not resolve such processes properly.

## 2. Results

### 2.1. Thrombocyte Imaging

**Figure 2:**
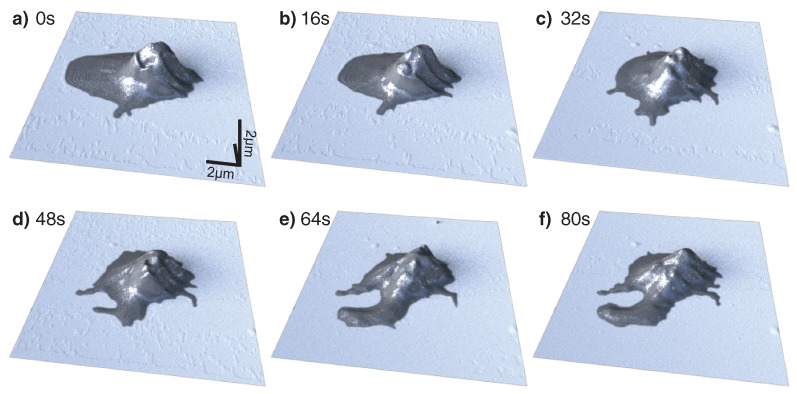
The attachment of a human thrombocyte onto a glass cover slip measured at 40 kHz photothermal off-resonance tapping AFM. Pseudopodia, which can be seen forming in (**a**), keep expanding (**c**–**f**) as the thrombocyte attaches to the surface. The shadow visible to the left of the cell in (**a**–**c**) is due to insufficient tip length and subsequent force interaction with the cantilever. Imaging rate is 16 s/frame, 4 lines/s.

Thrombocytes are a component in blood which are involved in the formation of clots that stop bleeding from wounds. They are nucleus-free cells produced in bone marrow [32,33]. Due to their relatively small size, in the order of 3–10 μm, they are a challenging sample for optical microscopy to resolve spatially [34]. Due to the large oscillation amplitudes in PORT and the subsequently low lateral forces, the initial adhesion and spreading of a thrombocyte can be observed (see Figure 2). In just a few seconds, the thrombocyte, which still retains most of its lentil shape, forms pseudopodia which act as additional anchors for the cell while it spreads on the glass surface.

**Figure 3:**
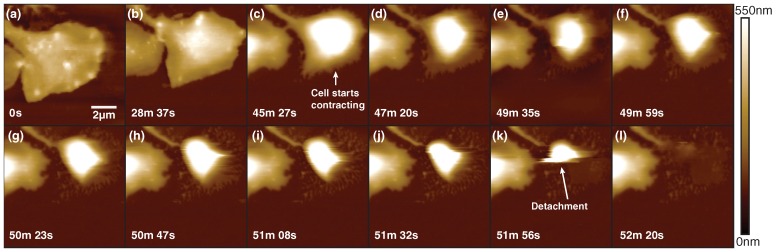
Time-lapse measurement of thrombocyte detachment from glass surface. While initially spread on the surface (**a**), the cell over time contracts (**b**) and changes into a more spherical shape (**c**), keeps increasing in height (**d**-**j**), then eventually detaches from the surface (**k**). After detachment, residual cell matter can be seen where the cell used to reside (**l**). Imaging rate is 23 s per frame, 11 lines/s.

In addition to short-term cell dynamics, like attachment, we have used PORT for time-lapse imaging on thrombocyte cells that are fully spread on a glass surface and subsequently detach (see Figure 3, Supplementary Movie S1).

Initially, the thrombocytes are mostly static (Figure 3a,b). Over time the cell starts contracting and gains in height appreciatively (Figure 3c,d). Over the next minute the cell continuously reduces the area which is in contact with the glass surface and returns to a more lentil-shaped form. During this process the membrane at the edges of the cells retracts, but patches of cellular matter are seen to stay on the glass. Eventually, the attachment of the cell to the glass surface is insufficient to hold the cell down. Subsequently, the thrombocyte is swiped away by the AFM tip. A thin layer of presumably membrane material remains where the cell was originally attached on the surface. The layer is spotty towards the borders of where the cell used to reside, and it is continuous in the center.

### 2.2. Bacterial Imaging

In order to show the suitability of PORT to imaging processes of bacterial cells, we imaged the membrane disruption of both a Gram-negative bacterium (*Escherichia coli*) as well as a Gram-positive bacterium (*Bacillus subtilis*).

We have previously shown that the antimicrobial peptide CM-15 causes surface roughening on *E. coli* cells [35,36]. Here we repeated this experiment using PORT. In contrast to our previous work, here we used a minimal growth medium, which prolongs cell viability significantly as compared to suspending them in deionized water. However, due to the ionic strength of the growth medium, the immobilization with poly-l-lysine is significantly weaker, and scanning the cells in amplitude modulation often causes them to detach from the surface [37]. Using PORT, we were able to scan live *E. coli* in unsupplemented as well as supplemented growth medium over extended periods of time. The time per experiment on live bacterial cells is generally limited by evaporation of the liquid or by formation of bubbles in the optical path. If care is taken that the fluid does not evaporate, experiments of more than 6 h are commonly achieved.

**Figure 4:**
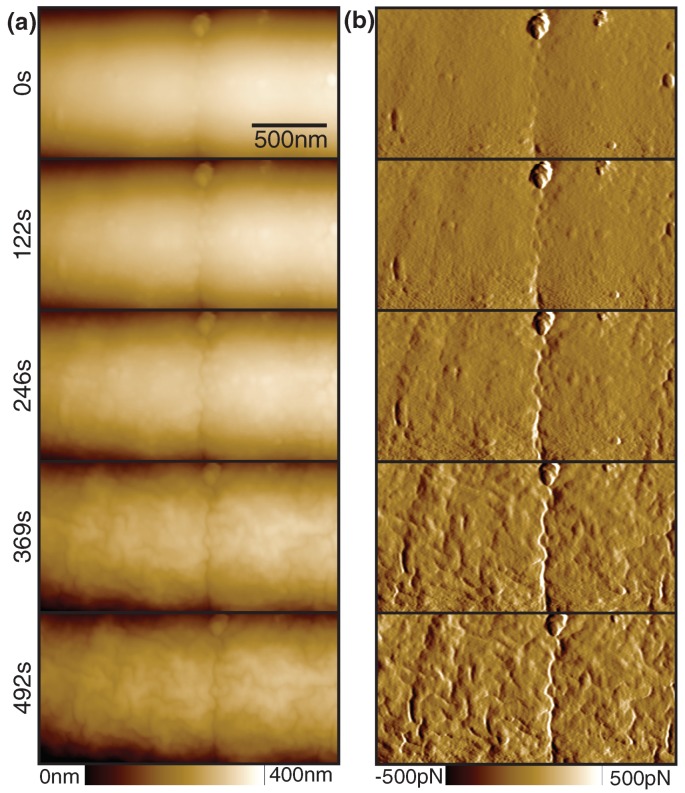
Membrane disruption on *E. coli* due to attack by the antimicrobial peptide CM-15 (added at *t* = 0 s). The cell membrane is significantly deformed over time. Notably, the central constriction where the cell has started dividing becomes more pronounced. (**a**) Height image. (**b**) Force error image. Images were taken at 12 s/frame, 10.3 lines/s, measured at 16 kHz PORT rate.

Figure 4 shows a time-lapse sequence in which CM-15 modifies the cell wall (see Supplementary Movie S2). Over time the cell seems to shrivel up while initially maintaining turgor pressure [38]. The effect is especially pronounced at the constriction where the cell has started dividing. A more pronounced buckling can be observed at the location of this constriction, which might result from the stress geometry of the constriction itself.

Compared to Gram-negative bacteria, the cell wall of Gram-positive bacteria, like *B. subtilis*, is significantly thicker, estimated to be about 30–60 nm thick, a large fraction of which is composed of a peptidoglycan layer that forms the outer cell wall [39]. This layer forms the load bearing support for the bacterial cell. We perturbed the peptidoglycan layer by treating cells with lysozyme, an enzyme that hydrolyzes cross-linked peptidoglycan. Lysosomal activity in eukaryotic internalized vesicles is an innate part of the mammalian immune reaction. Lysozyme hydrolyzes peptidoglycan, compromising bacterial structural integrity, causing morphological bulging [40], and ultimately causing bacterial lysis.

**Figure 5:**
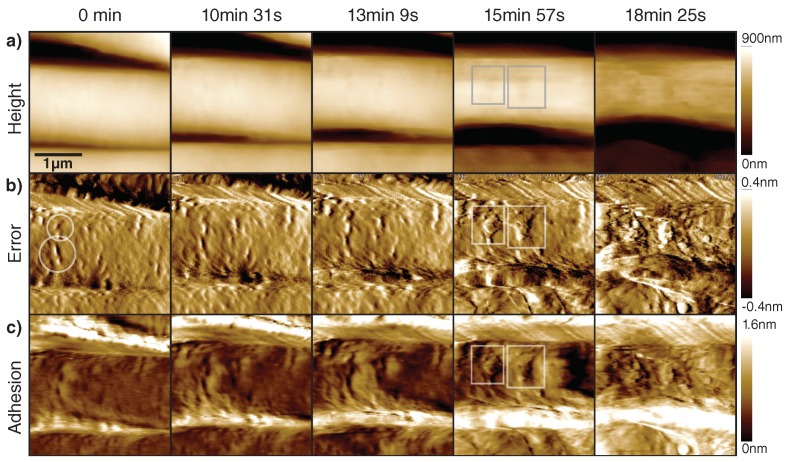
The effect of lysozyme on the cell wall of *B. subtilis* in growth medium. Lysozyme was added at *t* = 10 min. (**a**) Height image. (**b**) The error image shows initial scars in the cell wall (circled marks) of the bacterium, which then expand to patches (square marks) where the peptidoglycan layer is partially removed, followed by rapid loss of turgor pressure and subsequent cell death. (**c**) The PORT adhesion increases slightly as more of the peptidoglycan layer is hydrolyzed. Notable are dark patches visible in the height channel (square marks), which have lower adhesion where presumably most of the peptidoglycan has been stripped. Scan size 3 μm, 7 lines/s, measured at 25 kHz PORT rate.

We have acquired time-lapse AFM images of the lysis process on the cell wall of *B. subtilis* bacteria (see Figure 5, Supplementary Movie S3). After 10 min of imaging, we added a substantial amount of lysozyme (20 μL of 500 μg mL${}^{-1}$ lysozyme in 50 mM TRIS) to the liquid cell of the AFM. A few seconds after injection (*t* = 10 min), we see a slight roughening of the cell wall, together with a widening of already present scars on the outside of the bacterium (see Figure 5b, circled marks). An initial small decrease in cell diameter is also apparent, suggesting a loss of turgor pressure. The scars expand over the next few minutes, until whole patches of peptidoglycan are dissolved and the underlying layer is exposed (see Figure 5a, square marks). With ongoing dissolution of the peptidoglycan layer, the cell loses its structural integrity and deflates over the next minutes, until only a very soft remainder of the sacculus is present, which is readily displaced by the tip.

## 3. Discussion

Here, we have shown the advantages of using PORT for scanning live bacterial and eukaryotic cells. We have demonstrated intrinsic cell behaviors with the attachment and detachment of human thrombocyte cells, as well as reactions of cells due to externally induced factors.

The main benefit of PORT for live cell imaging is the ability to combine the high speed of resonant tapping with the large oscillation amplitudes that allow for a big periodic clearance from the sample, in turn reducing the lateral forces that often displace high, weakly bound samples. Additionally, mechanical information can be extracted from the measurement at no added complexity to the user (see Figure S1). Furthermore, the direct observability of the force interaction and the de facto immunity to changes in the tip and the cantilever—both of which are major problems in resonant tapping—making fully automated measurements possible, which enables investigations of problems that are not addressable with conventional amplitude modulation AFM.

As with any atomic force microscopy imaging, cantilever selection is crucial to achieving good results. The three most defining parameters for dynamic live cell imaging are the tip length, the spring constant, and the resonant frequency. Cantilever interactions due to insufficient tip length, such as those that are visible in Figure 2a–c, can lead to a wrong interpretation of height data and make volume calculations impractical. The spring constant and the resonant frequency are geometrically linked. As the spring constant determines the interaction force during the measurement, soft cantilevers are preferred. The miniaturization of cantilevers increases the measurement speed while keeping the spring constant low [41,42]. Focused electron beam-induced deposition (FEBID) of tips [43,44,45] can be used to grow micron-sized high aspect ratio tips on cantilevers that are supplied with short tips. We used FEBID to grow diamond-like carbon spikes of 4–1.5 μm length as tips on top of the pre-existing 800 nm high tip on our small cantilevers (Olympus BL-AC10-DS). Together, the total tip length is sufficient to provide enough clearance for most biological samples [46].

While PORT offers drastically improved feedback bandwidth compared to other ORT modes, it should be noted that achievable modulation amplitudes in liquid are usually limited to less than 150 nm. While this is more than sufficient for most samples, it might be insufficient to overcome sample adhesion forces in the case of a very adherent substrate, such as with polydimethylsiloxane (PDMS).

Even at the increased feedback speed, the large topography change which is especially pronounced at the border of a bacterial cell can cause the whole off-resonance amplitude to be quenched when scanning very fast. In this case, the resulting large lateral forces can still result in the displacement of the cell. Standard techniques, such as aligning the scan direction along the bacterium, should still be employed. Finally, using pyramidal tips is recommended for imaging bacteria, as high aspect ratio tips lead to sudden impacts with the cell.

The examples shown here are meant to demonstrate the versatility of the technique. Other examples of interesting dynamic processes include, but are not limited to thrombocyte migration, growth and division of morphologically different bacteria, pilus dynamics and mechanics, and endospore formation; it can further be used to study host–pathogen interactions. We believe that PORT opens up a large variety of possibilities in live cell imaging to study rapid behavior of live cells, and will continue the success of off-resonance AFM in cell biology.

## 4. Materials and Methods

### 4.1. Thrombocyte Preparation

Thrombocyte samples were prepared by pipetting 10–20 μL of fresh blood, harvested from a lancet puncture of a finger tip, onto a clean 12 mm glass cover slip glued on a magnetic steel sample disk. The drop of blood was left to incubate for 5 min for thrombocytes to form initial attachment. The cover slips were then washed in Tyrode’s Solution (3 mM HEPES, 137 mM NaCl, 4 mM NaH${}_{2}$PO${}_{4}$, 2.6 mM KCl, 1 mM MgCl${}_{2}$, and 1 mM glucose) to remove any non-attached blood components. After washing, samples were immediately transferred to the AFM for imaging. Glass cover slips were cleaned by ultrasonication in 9:1, 1:1, and 1:9 mixtures of chloroform:ethanol, 1 min each.

### 4.2. Bacteria Preparation

*E. coli* MG1665 cells were kindly donated by Yoshiko Miyahara from McKinney Lab at EPFL, and *B. subtilis* 168 WT cells were donated by Stephan Gruber from UNIL. Cells were inoculated in LB medium (*B. subtilis* and *E. coli* in Figure 4) or in enriched M9 minimal medium (*E. coli* in Figure 1e) in an orbital shaker at 37 ^∘^C and harvested in the exponential phase. *E. coli* suspensions were subsequently washed three times in non-enriched M9 medium; *B. subtilis* was washed in M9 supplemented with 2 mM MgSO${}_{4}$ and 0.4% glucose. Cleaned cultures were deposited on functionalized substrates. The functionalized surfaces were prepared by first ensuring a clean surface. Mica disks were cleaved with scotch tape, and 12 mm glass cover slips were cleaned by 1 min O${}_{2}$-plasma in a microwave plasma asher at 500 W (TePla 300). Both mica and glass surfaces were functionalized with poly-l-lysine (PLL) by immersing the substrate for half a minute in PLL solution (50 μg mL${}^{-1}$ PLL in 10 mM TRIS, pH 8), then wicking off the drop with a paper tissue, rinsing thoroughly in deionized water and drying with a nitrogen stream.

Cells in about 30 μL of solution were allowed to adhere to the surface between 5 min and 30 min, and were then rinsed in fresh buffer to remove floating cells. Prepared samples were immediately transferred to the AFM for imaging.

CM-15—a peptide with the sequence KWKLFKKIGAVLKVL—was bought from Genscript (Piscataway, NJ, USA) in lyophilized form and resuspended and diluted with Millipore water at 1 mg mL${}^{-1}$. CM-15 was added to the experiment to achieve a final concentration of 50 μg.

Lysozyme was bought from Sigma-Aldrich (L6876) in lyophilized form, then resuspended in 50 mM TRIS at a concentration of 5 mg mL${}^{-1}$ and diluted to the final concentration of 500 μg mL${}^{-1}$ before use.

### 4.3. AFM Imaging

All imaging was performed on a home-built atomic force microscope, based on the Bruker MultiMode. A custom-built drop-in replacement for the original microscope head, providing the means to use small cantilevers and photothermal excitation, was used and is described in detail elsewhere [28,29,30,47].

All imaging was done with either Olympus AC10DS (for thrombocytes) or Bruker FastScan-D cantilevers (for bacterial imaging). PORT amplitudes used were between 25 and 120 nm, with typical setpoints between 0.5 and 5 nm, corresponding roughly to between 50 and 2000 pN.

PORT for platelet and *E. coli* imaging was implemented using a Nanoscope 5 controller with a modified PeakForce-HR workspace that allows splitting the modulation from the *z*-signal. A custom-built scaling and offset circuit was used to adapt output voltage levels as required by the head electronics. *B. subtilis* imaging was performed using home-built AFM software based on a standalone FPGA (USB-7856R OEM, National Instruments, Austin TX, USA), with software and hardware programming as described elsewhere [30], and using the amplifiers in a modified Nanoscope-IIIa controller (Digital Instruments, Santa Barbara, CA, USA) that allow for input of external low-voltage scan signals. All images were acquired on a J-scanner (Bruker, Santa Barbara CA, USA) (120 × 120 × 5.2 μm range).

## Supplementary Materials

The following are available online at http://www.mdpi.com/1422-0067/19/10/2984/s1, Figure S1: Mechanical contrast extraction during a scan of a thin-film styrene-ethylene-butylene-styrene block copolymer in PORT.

## Abbreviations

The following abbreviations are used in this manuscript:

AFM	Atomic force microscopy
FPGA	Field Programmable Gate Array
HEPES	4-(2-hydroxyethyl)-1-piperazineethanesulfonic acid
PLL	Poly-l-lysine
PORT	Photothermal off-resonance tapping
TRIS	tris(hydroxymethyl)aminomethane
