# Supplementary material for: Photothermal Off-Resonance Tapping for Rapid and Gentle Atomic Force Imaging of Live Cells
Source: Int J Mol Sci. 2018 Sep 30;19(10):2984. doi: 10.3390/ijms19102984 (PMC6213139; doi:10.3390/ijms19102984)
Supplement: Supplementary file 1 [file ijms-19-02984-s001.zip › SI/nievergelt_ijms_2018_r1_SI.pdf]

Supplementary Materials: Photothermal off-resonance tapping for rapid and gentle atomic force imaging of live cells

Adrian P. Nievergelt [1], Charlène Brillard[1], Haig A. Eskandarian[1,2] John D. McKinney[2] and Georg E. Fantner[1*]

1. Mechanical contrast extraction in photothermal off–resonance tapping AFM

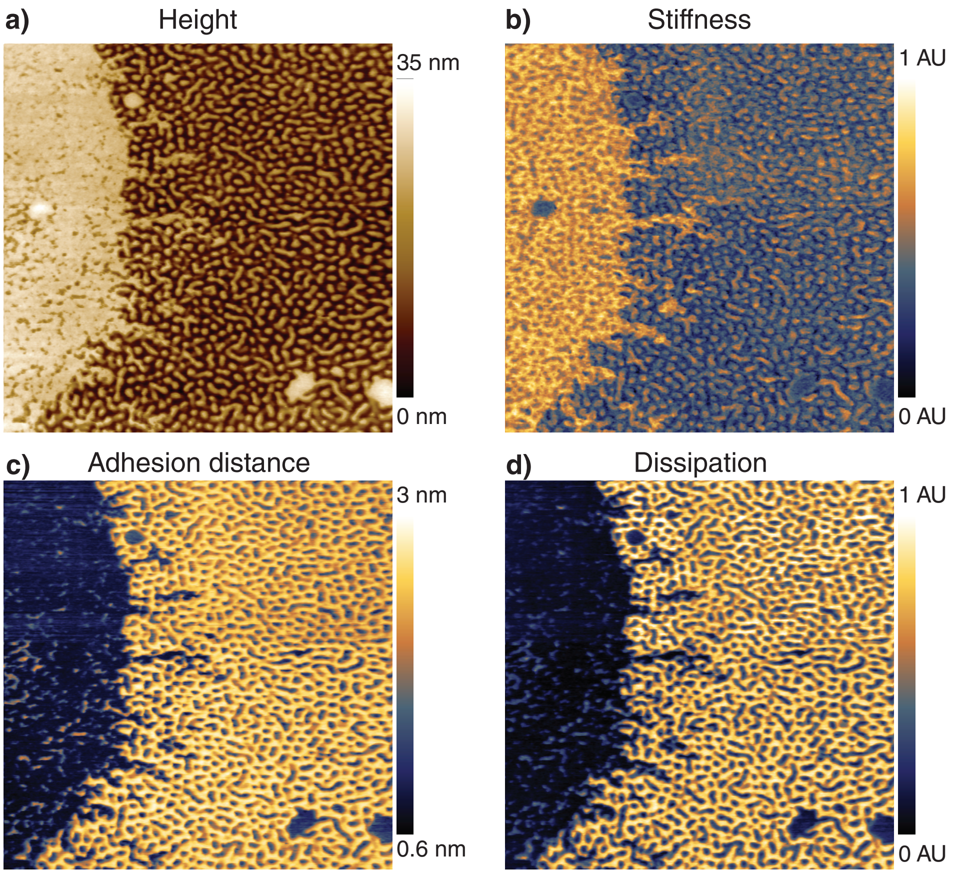

Figure S1. Simultaneous topography measurement and mechanical contrast extraction during a scan of a thin–film sample of Styrene–Ethylene–Butylene–Styrene (SEBS) block copolymer on silicon in PORT. Data channels shown are a) height information at peak force, b) stiffness, directly related to the young's modulus of the underlying sample, c) adhesion distance and d) dissipation or energy loss during an approach–retract cycle. Sample scanned at 2 lines/s in air at 10 kHz PORT rate with a Bruker FastScan-D cantilever.
