# Supplementary figures and images for: Photothermal Off-Resonance Tapping for Rapid and Gentle Atomic Force Imaging of Live Cells
Source: Int J Mol Sci. 2018 Sep 30;19(10):2984. doi: 10.3390/ijms19102984 (PMC6213139; doi:10.3390/ijms19102984)

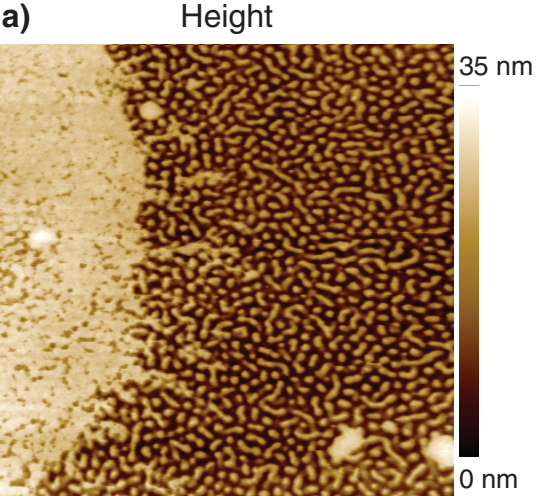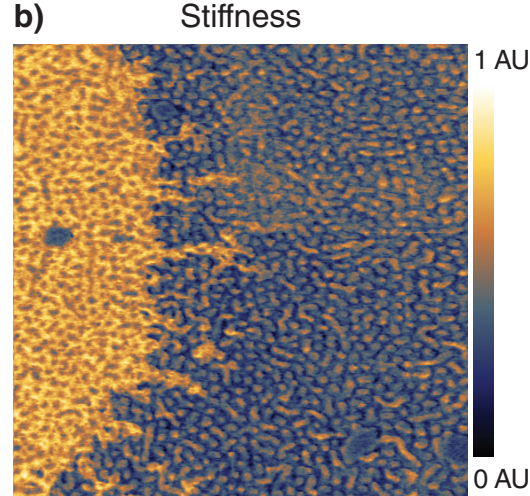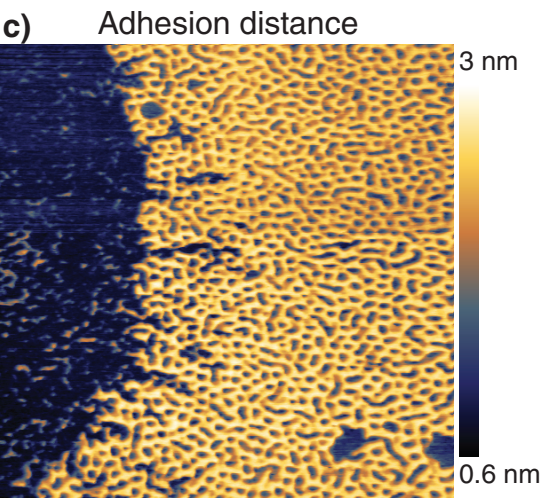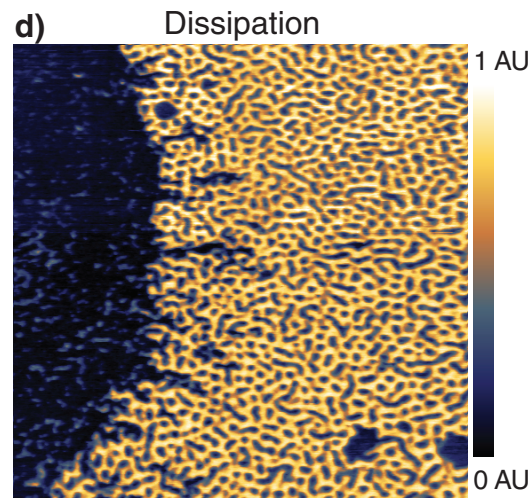

Supplement: Supplementary file 1 [file ijms-19-02984-s001.zip › SI/si_sebs.pdf]
